# Unraveling the proteomic signatures of coronary artery disease and hypercholesterolemia

**DOI:** 10.17305/bb.2024.10111

**Published:** 2024-10-24

**Authors:** Gulsen Guliz Anlar, Shona Pedersen, Sarah Al Ashmar, Hubert Krzyslak, Layla Kamareddine, Asad Zeidan

**Affiliations:** 1 Department of Basic Medical Sciences, College of Medicine, QU Health, Qatar University, Doha, Qatar; 2 Department of Clinical Biochemistry, Aalborg University Hospital, Aalborg, Denmark; 3 Department of Biomedical Science, College of Health Sciences, QU Health, Qatar University, Doha, Qatar; 4 Biomedical Research Center, Qatar University, Doha, Qatar

**Keywords:** Proteomics, coronary artery disease (CAD), hypercholesterolemia (HC), biomarkers, atherosclerosis

## Abstract

Atherosclerosis, a leading cause of coronary artery disease (CAD), is heavily influenced by hypercholesterolemia (HC). Proteomics research has shown promise in identifying biological markers for CAD diagnosis and prognosis. This cross-sectional study aimed to identify novel biomarkers for detecting HC and CAD. Through the analysis of proteome data from healthy controls (*n* ═ 45) and patients diagnosed with HC (*n* ═ 51) or CAD (*n* ═ 32), distinct protein patterns associated with each condition were identified. Significant alterations in protein levels were identified with a false discovery rate (FDR)-corrected *q* value of <0.05. Subsequent receiver operating characteristic (ROC) analysis, with an area under the curve (AUC) greater than 0.75, was conducted. CAD patients exhibited significantly increased levels of the cholesterol-metabolizing protein proprotein convertase subtilisin/kexin type 9 (PCSK9) and varied levels of the angiogenesis-related protein stromal-cell-derived factor-1 (SDF-1) compared to controls. In pairwise comparisons among the study groups, 65 proteins showed significant differential expression. Notably, 14 of these proteins had significant correlations with blood cholesterol levels. Additionally, 22 of the identified proteins were associated with CAD or HC pathways, with nine proteins being common to both conditions (APO E, APO E3, MMP-3, PCSK9, SDF-1, APO B, PAFAH, 60 kDa heat shock protein, and TGF-beta-activated kinase 1 and MAP3K7-binding protein 1 fusion). Nevertheless, this is an exploratory study, and validation studies are needed to confirm these potential protein biomarkers for CAD in the context of HC.

## Introduction

Cardiovascular disease (CVD) is the leading cause of mortality and a significant cause of morbidity worldwide [1]. Coronary artery disease (CAD) is the most common type of CVD [1], causing the narrowing of blood vessel lumens due to atherosclerotic plaque formation, which increases the risk of life-threatening events, such as heart attacks and heart failure [2]. Hypercholesterolemia (HC) is characterized by elevated cholesterol levels in the blood [3]. High lipid levels can accumulate in arterial walls, raising the risk of CVD [3]. Cholesterol is transported in the blood via lipoproteins, with two main types: high-density lipoprotein (HDL), known as “good” cholesterol, and low-density lipoprotein (LDL), also known as “bad” cholesterol [4]. The majority of cholesterol found in atherosclerotic plaques is derived from LDL [5], making a sustained increase in plasma LDL cholesterol a major factor in the development of atherosclerosis [6]. Thus, investigating molecular changes in HC is crucial to understanding the fundamental mechanisms behind its complications.

Non-invasive imaging techniques, such as coronary artery calcium (CAC) scoring and computed tomography (CT) angiography, have been shown to improve risk prediction and classification compared to conventional risk variables alone by detecting coronary calcification or plaques [7]. Current guidelines (2021 ESC guidelines) consider the CAC score and carotid plaque identification as risk modifiers in cardiovascular risk evaluation, particularly for individuals whose calculated cardiovascular risks are near decision thresholds based on primary conventional risk factors [8].

However, routine screening for atherosclerosis using imaging modalities raises concerns about accessibility, cost-effectiveness, required expertise, and radiation exposure. Consequently, the idea of using circulating biomarkers to enhance cardiovascular risk estimation is gaining traction. Various biomarkers, including high-sensitivity C-reactive protein, lipoprotein(a), natriuretic peptides, and high-sensitivity cardiac troponin, have been proposed to improve risk classification. Moreover, studies have shown that incorporating polygenic risk scores (PRS) with these biomarkers can improve coronary heart disease (CHD) prediction and help identify high-risk populations, enabling individuals with the highest PRS values to benefit from early prevention efforts [9–11].

The ability to measure and interpret endogenous protein profiles from biological samples has opened new avenues for researchers to investigate biological variations among subpopulations, whether due to drug therapy, disease, or morbidity. This has led to a more comprehensive understanding of these processes [12]. Proteomics studies, in particular, offer opportunities to discover novel protein biomarkers directly associated with atherosclerosis occurrence, progression, and complications, such as plaque instability or rupture [13, 14]. Research on HC patients and animal models using proteomics has assessed treatment responses and the progression of complications [15, 16].

Despite substantial research and advancements in CVD treatment, it remains a global burden with a high fatality rate. Given the severe complications associated with CVD, prevention or at least early detection of atherosclerotic disease can help save lives. HC is a leading risk factor for CAD, making it essential to understand the mechanisms driving HC patients toward CAD to develop more effective preventative approaches. This study aimed to identify specific protein changes in individuals with HC compared to those with CAD using quantitative proteomics, with the ultimate objective of identifying potential clinical markers for CAD.

## Materials and methods

### The study population

This cross-sectional study included 45 healthy controls (Con) with no chronic disease, 32 patients with CAD, and 51 patients with hypercholesterolemia (HC). All participants met the inclusion and exclusion criteria and had available proteomics data. The inclusion criteria were as follows:

For the control group included healthy adults; for HC group included adults with LDL cholesterol levels above 3.36 mmol/dL (130 mg/dL); and for CAD group included adults with a self-reported previous diagnosis of angina or heart attack. The exclusion criteria for the control and HC groups included blood hemoglobin A1c (HbA1c) levels above 6.4%, systolic blood pressure above 139 mmHg, or diastolic blood pressure above 89 mmHg.

Sample collection, processing, biochemical and biometric analysis, as well as clinical and proteomic data provision were carried out by Qatar Biobank (QBB).

### Sample collection and processing prior to proteomic analysis

The collection of biological specimens has been previously described in detail [17]. Sixty milliliters of blood were drawn from each participant. Routine clinical indicators were measured by analyzing a portion of the blood. The remaining serum samples were divided into multiple aliquots, which were then placed in microtubes. These sample aliquots were either stored at −80 ^∘^C for immediate use or in liquid nitrogen for long-term storage. EDTA plasma, buffy coat (leukocytes), and erythrocytes were separated by centrifuging the blood samples.

### Biochemical and biometric analysis

Biochemical biomarkers, including complete blood count, liver and kidney function tests, blood glucose, thyroid hormone, and C-reactive protein levels, were assessed by collecting blood samples from participants. The hematology and blood biochemistry analyses were conducted at the laboratory in Doha’s Hamad Medical Center [17]. Each participant’s weight and height were measured using a Seca stadiometer (Tanita). Arterial stiffness was evaluated by measuring pulse wave velocity (PWV) with the VICORDER device (Skidmore Medical, U.K.). Diastolic and systolic blood pressure readings were taken twice using the automated Omron 705 device; a third reading was taken if there was a discrepancy of at least 5 mmHg between the initial readings.

### Targeted proteomics profiling by modified aptamer (SOMAmer)-based protein array

Proteomics profiling was conducted at Weill Cornell Medicine-Qatar using a slow off-rate modified aptamer (SOMAmer)-based protein array (SomaLogic, Boulder, CO, USA), as previously described [18, 19]. Plasma samples were diluted and treated with aptamers that selectively target bead-coupled epitopes (SOMAmers). Biotinylated proteins were then attached to the beads. After photocleavage, complexes of biotinylated target proteins and fluorescence-labeled SOMAmers were captured on streptavidin beads. The SOMAmers were subsequently eluted and quantified by hybridizing them to specific arrays of complementary oligonucleotides.

The resulting raw intensities were processed using multiple reference standards, which included signal calibration, median signal normalization, and hybridization normalization to account for inter-plate variations. Proteomics data was presented in relative fluorescence units (RFUs), with no missing values in the dataset.

### Ethical statement

The study was conducted according to the guidelines of the Declaration of Helsinki, and approved by the Institutional Review Board of Qatar Biobank, Doha, Qatar (IRB protocol no: E-2021-QF-QBB-RES-ACC-00022-0163).

### Statistical analysis

Demographic and clinical information were presented as means with standard deviations (mean ± SD) using descriptive statistics. Statistical significance of the parameters was determined with a *P* value < 0.05 using ANOVA for the three study groups, followed by Tukey’s multiple comparisons test. GraphPad Prism 9.2.0 (La Jolla, CA, USA) was used for statistical analysis of patient demographics.

Before proteomics data analysis, RFU values were log-transformed to ensure a normal distribution. Then, unsupervised principal component analysis (PCA) was conducted, followed by orthogonal partial least squares discriminant analysis (OPLS-DA) to identify orthogonal components that best differentiated between sample groups. PCA and OPLS-DA were performed with SIMCA version 16 (Umetrics, Umeå, Sweden).

For proteomics data analysis, multiple statistical methods and selection criteria were applied. Pairwise comparisons were conducted between CAD, HC, and control groups (CAD vs control, HC vs control, and CAD vs HC). A Student’s *t*-test was used to identify proteins significantly differentially expressed between groups. Proteins with a *P* value < 0.05 were considered significant and selected for further analysis. Differentially expressed proteins were visualized as a heatmap after z-scaling, while volcano plots illustrated log2 fold change (FC) values for these proteins.

Next, multiple testing correction for *P* value adjustment was performed using the Benjamini–Hochberg method, with *q*-values calculated through false discovery rate (FDR) estimation. Proteins with a *q* value < 0.05 were considered significantly altered between groups and subjected to further analysis. Additionally, receiver operating characteristic (ROC) analysis was conducted to determine the area under the curve (AUC), sensitivity, and specificity of the proteins. Proteins with AUC > 0.75 were considered as significant.

In summary, a protein’s expression was considered statistically significant if it met three criteria: *P* value < 0.05, and its *q* value < 0.05, and its AUC > 0.75. Finally, correlation analysis was conducted between proteins with statistically significant expression and blood lipid levels. Z-scaling, clustering, Student’s *t*-tests, and Benjamini–Hochberg methods were performed with Perseus 1.6.13.0. GraphPad Prism 9.2.0 (La Jolla, CA, USA) software was used to generate heatmaps, volcano plots, box plots, ROC curves, and correlation analyses. Raw RFU values were used as inputs for box plots and ROC curves.

Pathway analysis was performed using the Kyoto Encyclopedia of Genes and Genomes (KEGG) and gene–disease associations (DisGeNET) databases to gain further insight into the biochemistry of the identified proteomes. This analysis was conducted using the GeneCodis platform (accessible at https://genecodis.genyo.es/).

## Results

### Characteristics of study populations

This study included 128 participants divided into three distinct health condition groups: CAD (*n* ═ 32), HC (*n* ═ 51), and healthy controls (*n* ═ 45). Participants across all groups ranged in age from 24 to 67 years, although the control group was, on average, slightly younger than both disease groups. PWV was measured to assess arterial stiffness. PWV values between 10 and 12 m/s are considered borderline, while values exceeding 12 m/s strongly indicate arterial rigidity. The HC group had a mean PWV of 12.75 m/s, and the CAD group had a mean PWV of 14.97 m/s, aligning with expectations; HC patients are at risk for developing atherosclerotic diseases, and CAD patients typically already experience arterial stiffness.

All groups were classified as overweight based on BMI, with minor distinctions between control and disease groups. Despite significant *P* values in group comparisons, systolic and diastolic blood pressure levels remained within the normal range for all participants. Similarly, although white blood cell count distributions showed a significant *P* value between groups, cell counts did not exceed the maximum reference level.

In the HC group, HDL cholesterol levels were lower, while total cholesterol (TC), LDL cholesterol, and triglyceride (TG) levels were elevated, indicating dyslipidemia. Blood count parameters, as well as liver and kidney function indicators, did not differ significantly among the three groups (Table 1). More detailed data with pairwise group comparisons are provided in Table S1, and information on participants’ medication use can be found in Table S2.

**Table 1:** Participant characteristics of the study groups

	**Control** **(*n* ═ 45)**	**HC** **(*n* ═ 51)**	**CAD** **(*n* ═ 32)**	**Reference range**	***P* value**
Gender (Female/Male)	24/21	26/25	14/18	-	
Age	42.44 (6.2)	49.33 (10.1)	50.5 (11.2)	-	0.0002
PWV (m/s)	9.94 (1.8)	12.75 (3.4)	14.97 (6.3)	<12	<0.0001
BMI (kg/m^2^)	27.31 (4.3)	30.91 (5.5)	30.93 (5.9)	18.5–24.9	0.0016
Systolic BP (mmHg)	109 (9.5)	116.2 (11.5)	122.8 (12.6)	90–139	<0.0001
Diastolic BP (mmHg)	71.09 (6.1)	76.57 (7.04)	75.53 (9.5)	60–89	0.0014
Hemoglobin (g/dL)	13.06 (2.4)	13.84 (1.7)	13.41 (1.5)	13–17	0.1672
RBC (×10^6^/uL)	4.858 (0.6)	4.84 (0.5)	4.90 (0.5)	4.5–5.5	0.8938
WBC (×10^3^/uL)	6.44 (1.7)	6.35 (1.8)	7.58 (2.06)	4.0–10.0	0.0084
Platelet (×10^3^/uL)	248.8 (77.5)	243.3 (71.1)	240 (66.7)	150–400	0.8644
Urea (mmol/L)	4.7 (1.4)	4.50 (1.4)	4.85 (1.3)	1.7–8.3	0.5245
Creatinine (umol/L)	65.22 (14.1)	70.27 (15.4)	72.97 (17.4)	53–124	0.0823
ALT (U/L)	19 (8.5)	24.49 (12.4)	23.25 (15.8)	0–40	0.0826
AST (U/L)	17.56 (4.3)	19.92 (6.1)	20 (9.5)	0–40	0.1555
HDL (mmol/L)	1.45 (0.3)	1.26 (0.2)	1.24 (0.3)	>1.55	0.0085
LDL (mmol/L)	2.56 (0.5)	4.14 (0.4)	2.65 (1.03)	<3.36	<0.0001
TG (mmol/L)	1.22 (0.8)	1.90 (1.08)	1.63 (0.9)	<1.69	0.0034
TC (mmol/l)	4.58 (0.6)	6.15 (0.6)	4.61 (1.1)	<5.2	<0.0001
TSH (mIU/L)	1.59 (1.04)	2.07 (1.3)	1.68 (1.2)	0.45–4.5	0.1189

Values are presented as mean and standard deviation. CAD: Coronary artery disease; PWV: Pulse wave velocity; BMI: Body mass index; BP: Blood pressure; RBC: Red blood cell; WBC: White blood cell; ALT: Alanine aminotransferase; AST: Aspartate aminotransferase; HDL: High-density lipoprotein; LDL: Low-density lipoprotein; TG: Triglyceride; TC: Total cholesterol; TSH: Thyroid stimulating hormone; HC: Hypercholesterolemia.

### Proteomic profiling of CAD and HC

**Figure 1.**
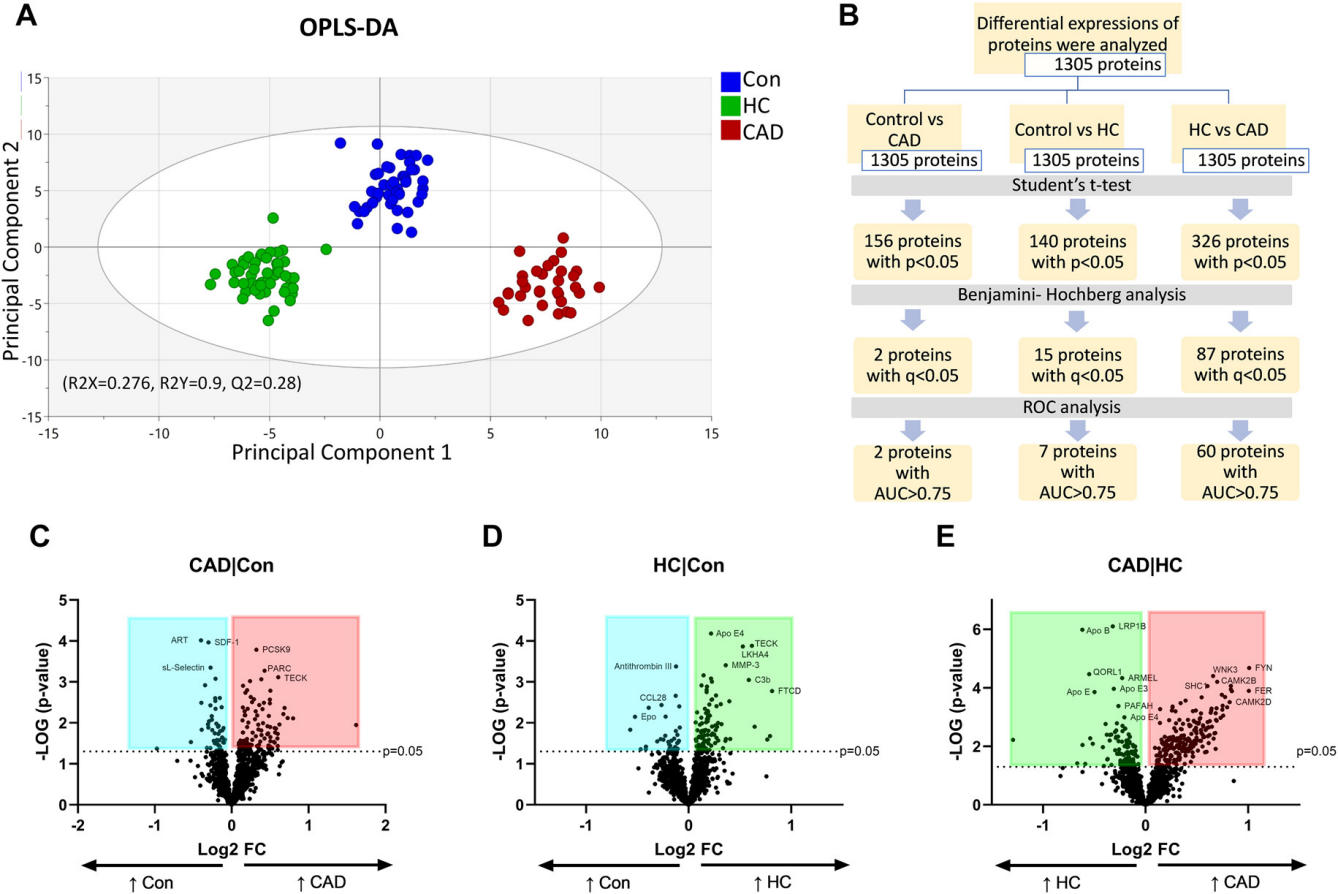
**Separation of study groups and differential protein expressions among Con, HC, and CAD groups.** (A) The graph illustrates the OPLS-DA separation of Con (blue), HC (green), and CAD (red); (B) The diagram summarizes the identification of significant proteins with statistical analyses; (C–E) Volcano plots depict differentially expressed proteins in multiple comparisons of CAD vs Con (C), HC vs Con (D), and CAD vs HC (E). Log_2_ FC indicates a fold increase (+) or decrease (−) between groups. Proteins with increased expressions in the CAD group (red), increased expressions in the HC group (green), and upregulated proteins in the control group (blue) are shown. Con: Control; HC: Hypercholesterolemia; CAD: Coronary artery disease; OPLS-DA: Orthogonal partial least square discriminant analysis; FC: Fold change.

The distribution of participants with proteomics data was assessed using PCA, and no outliers were observed (Figure S1). OPLS-DA revealed a clear separation of the three study groups based on two principal components, with R2X ═ 0.267, R2Y ═ 0.9, and Q2 ═ 0.28, indicating distinct protein alterations between the groups (Figure 1A). Multiple statistical analyses were then applied to the proteomics dataset to identify potential biomarkers for the disease groups. As summarized in Figure 1B, a total of 1305 proteins were analyzed, with 156 proteins showing significant differential expression in CAD vs control, 140 in HC vs control, and 326 in CAD vs HC comparisons, were significantly differentially expressed with a *P* value < 0.05.

**Table 2:** Top ten significantly differentially expressed proteins between Con, HC, and CAD groups

**CAD|Con**						
**UniProt ID**	**Gene name**	**Protein ID**	**Protein name**	**Log_2_ FC**	***P* value**	***q* value**
P48061	CXCL12	SDF-1	Stromal cell-derived factor 1	−0.30	0.0001	0.03
Q8NBP7	PCSK9	PCSK9	Proprotein convertase subtilisin/kexin type 9	0.32	0.0001	0.04
**HC|Con**						
**UniProt ID**	**Gene name**	**Protein ID**	**Protein name**	**Log_2_ FC**	***P* value**	***q* value**
Q9NZR2	LRP1B	LRP1B	Low-density lipoprotein receptor-related protein 1B	0.41	<0.0001	<0.0001
P02649	APO E	APO E	APOlipoprotein E	0.66	<0.0001	<0.0001
P04114	APO B	APO B	APOlipoprotein B	0.53	<0.0001	0.0008
P02649	APO E	APO E3	APOlipoprotein E (isoform E3)	0.35	<0.0001	0.001
O95825	CRYZL1	QORL1	Quinone oxidoreductase-like protein 1	0.60	<0.0001	0.001
P02787	TF	Transferrin	Serotransferrin	−0.16	<0.0001	0.002
P02649	APO E	APO E4	APOlipoprotein E (isoform E4)	0.22	<0.0001	0.005
P09960	LTA4H	LKHA4	Leukotriene A-4 hydrolase	0.53	0.0001	0.008
O15444	CCL25	TECK	C–C motif chemokine 25	0.62	0.0001	0.01
P01008	SERPINC1	Antithrombin-III	Antithrombin-III	−0.12	0.0004	0.028
**CAD|HC**						
**UniProt ID**	**Gene name**	**Protein ID**	**Protein name**	**Log_2_ FC**	***P* value**	***q* value**
P04114	APO B	APO B	APOlipoprotein B	−0.62	<0.0001	<0.0001
Q9NZR2	LRP1B	LRP1B	Low-density lipoprotein receptor-related protein 1B	−0.32	<0.0001	<0.0001
P06241	FYN	FYN	Tyrosine-protein kinase fyn	1.01	<0.0001	0.005
O95825	CRYZL1	QORL1	Quinone oxidoreductase-like protein 1	−0.55	<0.0001	0.006
Q49AH0	CDNF	ARMEL	Cerebral dopamine neurotrophic factor	−0.23	<0.0001	0.008
Q9BYP7	WNK3	WNK3	Serine/threonine-protein kinase WNK3	0.66	<0.0001	0.008
Q13554	CAMK2B	CAMK2B	Calcium/calmodulin-dependent protein kinase type II subunit beta	0.70	<0.0001	0.008
P29353	SHC1	SHC1	SHC-transforming protein 1	0.60	<0.0001	0.008
P15498	VAV1	VAV	Proto-oncogene vav	0.84	0.0001	0.009
P02649	APO E	APO E	APOlipoprotein E	−0.50	0.0001	0.01

A Log_2_ FC indicates a fold increase (+) or decrease (−) of the protein expressions in the first group compared to the second group. *P* < 0.05 and *q* < 0.05 are considered significant. Con: Control; HC: Hypercholesterolemia; CAD: Coronary artery disease; FC: Fold change.

**Figure 2.**
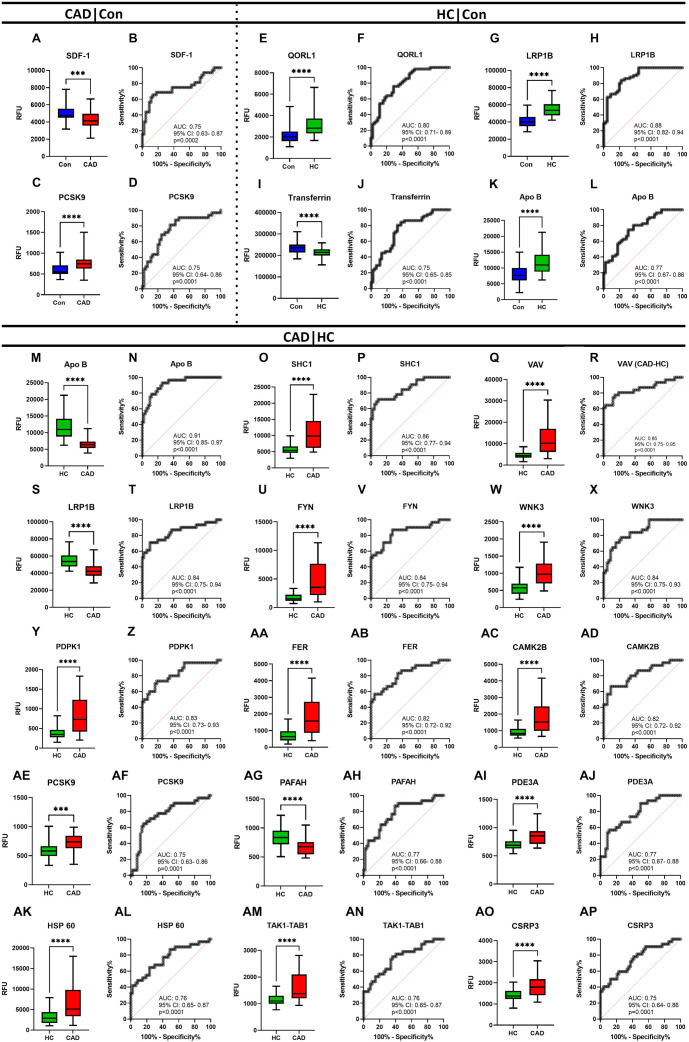
**Significantly differentially expressed proteins and their diagnostic values in Con, HC, and CAD groups.** Box plots represent differential expressions of proteins, and ROC curves show AUC values of differentially expressed proteins in pairwise comparisons of (A–D) CAD vs Con, (E–L) HC vs Con, or (M–AP) CAD vs HC. In CAD vs Con comparison, box plots of proteins are as in (A) SDF-1, (C) PCSK9. ROC curves are in B and D, respectively. In HC vs Con comparison, box plots of proteins are as in (E) QORL1, (G) LRP1B, (I) Transferrin, and (K) APO B. ROC curves are in F, H, J, and L, respectively. In CAD vs HC comparison, box plots of proteins are as in (M) APO B, (O) SHC1, (Q) VAV, (S) LRP1B, (U) FYN, (W) WNK3, (Y) PDPK1, (AA) FER, (AC) CAMK2B, (AE) PCSK9, (AG) PAFAH, (AI) PDE3A, (AK) HSP 60, (AM) TAK1-TAB1, and (AO) CSRP3. ROC curves are N, P, R, T, V, X, Z, AB, AD, AF, AH, AJ, AL, AN, and AP, respectively. *P < 0.05, ** *P* < 0.01, *** *P* < 0.001 **** *P* < 0.0001. All proteins in represented comparisons also have significant *q* values (<0.05). Con: Control; HC: Hypercholesterolemia; CAD: Coronary artery disease; ROC: Receiver operating characteristic; AUC: Area under the curve; SDF-1: Stromal-cell-derived factor-1; PCSK9: Proprotein convertase subtilisin/kexin type 9; LRP1B: LDL receptor-related protein 1B; VAV: Proto-oncogene vav; PDE3A: Phosphodiesterase 3A; HSP 60: 60 kDa heat shock protein; TAK1-TAB1: TGF-beta-activated kinase 1 and MAP3K7-binding protein 1 fusion; CSRP3: Cysteine and glycine-rich protein 3; QORL1: Quinone oxidoreductase-like protein 1; APO B: APOlipoprotein B; SHC1: SHC-transforming protein 1; FYN: Tyrosine-protein kinase Fyn; WNK3: Serine/threonine-protein kinase WNK3; CAMK2B: Calcium/calmodulin-dependent protein kinase type II subunit beta.

Protein upregulation and downregulation were evaluated in pairwise comparisons between CAD vs control (Figure 1C), HC vs control (Figure 1D), and CAD vs HC (Figure 1E), quantified as FCs and visualized in volcano plots (Figure 1C–1E). Specific details are provided in Table S2. After *P* value adjustment, two proteins with *q* < 0.05, exhibited significantly differential expression in CAD patients compared to healthy controls (Table 2). In contrast, HC patients showed 15 significantly altered proteins with increased expression (*q* < 0.05) compared to controls. Additionally, a comparison between CAD and HC patients identified 87 significantly regulated proteins (*q* < 0.05). All proteins with significant *P* and *q*-values are listed in Table S3, and the top ten significant proteins are presented in Table 2. Representative box plots of significantly differentially expressed proteins are displayed in Figure 2.

### Potential biomarker candidates differentiating CAD from HC

ROC analysis was performed on proteins with distinct expressions (*q* < 0.05) in pairwise group comparisons (Table S4) to identify potential biomarker candidates for distinguishing between the disease groups. AUC values were calculated, with an AUC between 0.75 and 90 considered to indicate excellent diagnostic value [20]. In the CAD vs control analysis, both significant proteins had an AUC of 0.75. In the HC vs control comparison, 7 out of the 15 significantly altered proteins showed an AUC of 0.75 or higher. Among the 87 proteins significantly differentially expressed in CAD vs HC, 59 had an AUC ≥ 0.75, with 16 of these proteins showing an AUC ≥ 0.80 (Table S4). Representative ROC curves for significantly differentially expressed proteins are shown in Figure 2.

### Proteomes related to lipid profile

**Figure 3.**
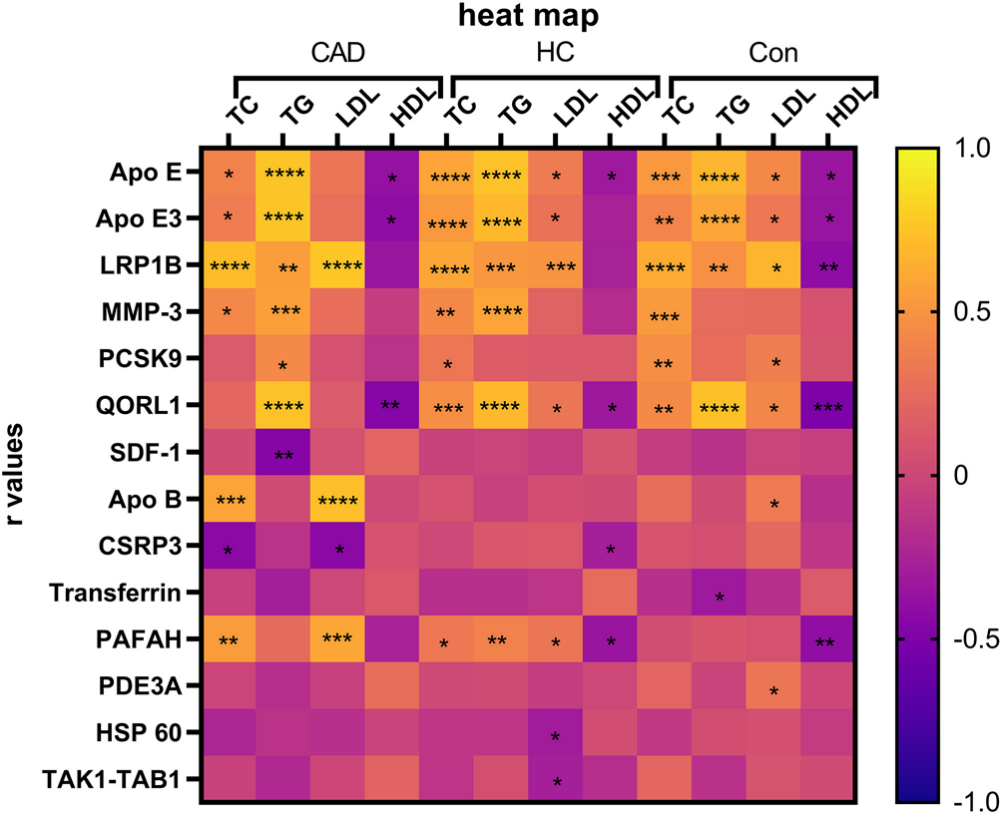
**Positive or negative correlation between protein levels and lipid profiles.** The heat map represents the *r*-values of each Pearson correlation analysis between lipid parameters and proteomes. *r* values are between +1 and −1. Statistically significant correlations were *P < 0.05, **P < 0.01, ***P < 0.001 ****P < 0.0001.

Proteins with an AUC ≥ 0.75 were selected for correlation analysis. Figure 3 shows the relationships between blood lipid parameters and regulated proteins for each comparison. In healthy control subjects, a significant correlation was found between APO E, APO E3, LDL receptor-related protein 1B (LRP1B), and QORL1 with all lipid parameters. Matrix metalloproteinase-3 (MMP-3) was significantly associated only with TC in this group, while proprotein convertase subtilisin/kexin type 9 (PCSK9) correlated with both TC and LDL. In the HC group, significant correlations were observed between APO E, QORL1, PAFAH, and all lipid parameters. However, APO E3 and LRP1B did not show significant correlations with HDL. MMP-3 was associated only with TC and TG in the HC group.

In CAD patients, APO E, APO E3, LRP1B, MMP-3, PCSK9, stromal-cell-derived factor-1 (SDF-1), and QORL1 were all significantly correlated with TG levels. Interestingly, 60 kDa heat shock protein (HSP 60) and TGF-beta-activated kinase 1 and MAP3K7-binding protein 1 fusion (TAK1-TAB1) showed a significant negative correlation with serum LDL levels only in the HC group. The correlation between SDF-1 and TG was specific to the CAD group. The relationships between transferrin and TG, and between phosphodiesterase 3A (PDE3A) and LDL, were significant only in the control group. MMP-3 displayed a significant positive correlation with TC across all groups and with TG in both CAD and HC groups. All significant correlations between proteins and blood lipids are listed in Table S5. Additionally, a significant negative correlation between the LDL receptor (LDLR) and PCSK9 was found only in the control group (Table S6).

### Pathway analysis

**Figure 4.**
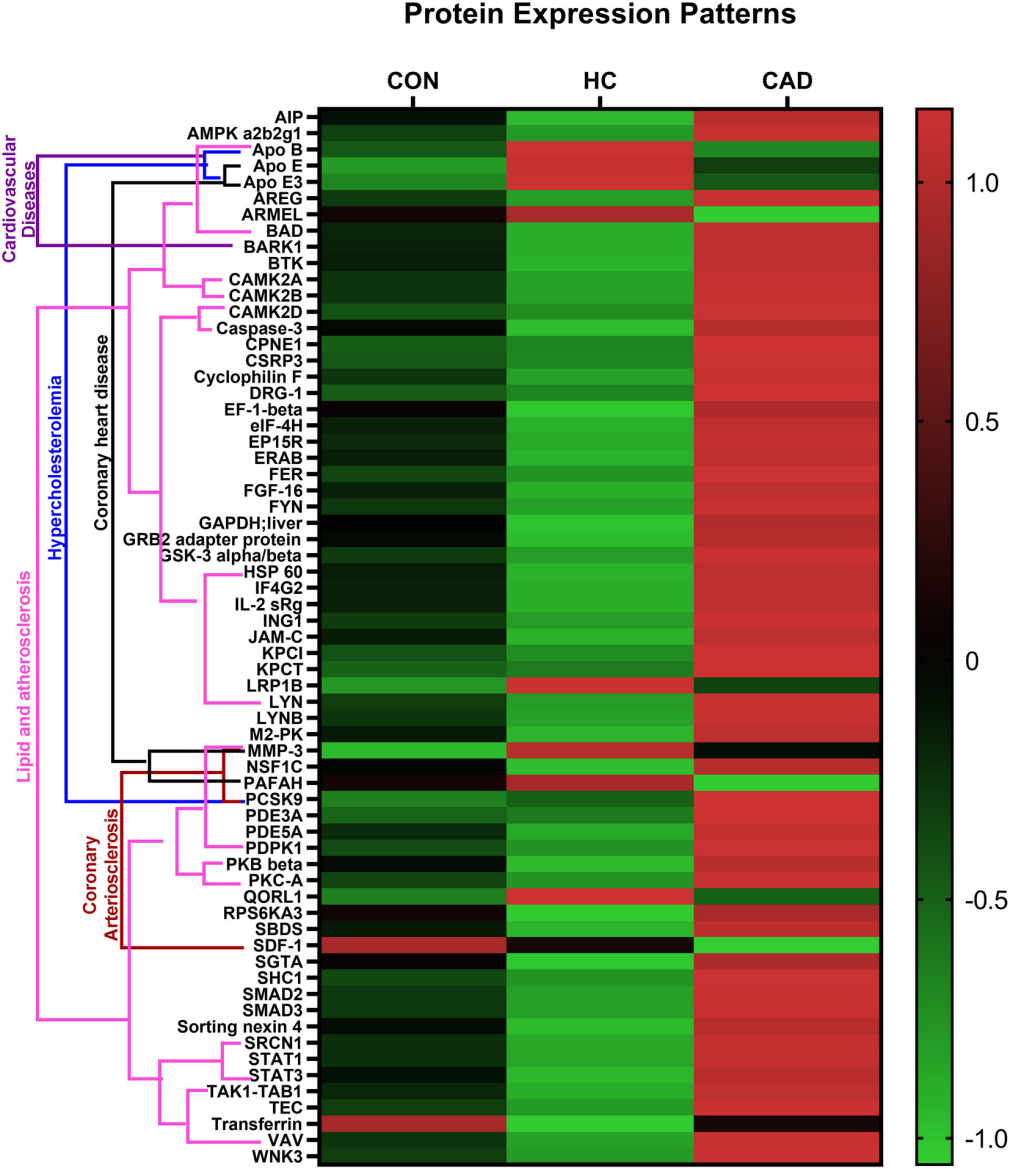
**Protein expression patterns and related pathways.** The figure shows significant protein up or down regulations among the study groups and their relation to disease pathways.

Proteins with significant *q*-values (<0.05) and AUCs (≥0.75) of ROC curves were included in pathway analysis using KEGG terms and the gene–disease association database DisGeNET. A total of 65 proteins were analyzed, of which 22 were enriched in pathways related to atherosclerotic diseases, with enrichment scores (ESs) ranging from 11 to 30. Additionally, three proteins—APO B, APO E, and PCSK9—were associated with hypercholesterolemia, showing an ES of 13.8 (Table 3). Most of the enriched proteins showed increased expression in the CAD group, except for APO B, APO E, APO E3, SDF-1, and PAFAH (Figure 4).

## Discussion

Proteomics research in this study identified potential biomarkers for CAD. Analysis of 1305 proteins revealed distinct profiles in control, HC, and CAD groups, particularly in cholesterol metabolism and inflammation-related pathways.

HC plays a critical role in atherosclerosis, which leads to CAD. High levels of LDL cholesterol diffuse from the bloodstream into the intimal layer of the arterial wall, where they are subsequently oxidized by free radicals. This oxidation results in the accumulation of oxidized LDL (ox-LDL) in the arterial wall, initiating the development of atherosclerotic plaque [21].

One protein identified with a significant increase in CAD patients was PCSK9, which plays a key role in cholesterol metabolism [22]. PCSK9 binds to LDLRs on liver cells, which are responsible for removing LDL cholesterol from the bloodstream by transporting it to the liver for degradation. When PCSK9 binds to LDLR, it reduces the recycling of LDLR back to the cell surface and enhances its degradation, resulting in fewer functional LDLRs available to clear LDL cholesterol from circulation. Consequently, elevated levels of PCSK9 may contribute to atherosclerosis by reducing LDL cholesterol clearance [22]. PCSK9 inhibitors are therefore recommended for managing dyslipidemia [8].

The study investigated the correlation of PCSK9 with lipid levels, finding an association with LDL (Figure 3) and LDLR (Table S6) in the control group, but not in the CAD or HC groups. This suggests a potential role for PCSK9 beyond cholesterol elevation in atherosclerosis. Emerging evidence supports this hypothesis, indicating that PCSK9 is associated with the necrotic core of atherosclerotic plaques in arterial walls, independent of serum LDL cholesterol levels [23]. Altogether, this suggests that PCSK9 inhibitors may have additional benefits for atherosclerosis treatment beyond reducing LDL cholesterol.

Another protein with a role in cholesterol metabolism is LRP1B, which aids liver cells in absorbing LDL [24]. Although several studies have examined LRP1B’s role in various cancers, its involvement in HC and CAD remains underexplored. However, one study reported that LRP1B SNPs were associated with cholesterol levels and CVD risk factors [25]. Additionally, an *in vivo* study showed that LRP1B inhibition leads to increased migration and invasion activities in vascular smooth muscle cells (VSMCs) in the atherosclerotic aorta [26]. LRP1B was also found to interact with ligands involved in coagulation, lipoprotein metabolism, cell adhesion, and immunity [27]. Together, these studies suggest that LRP1B may play a role in atherosclerosis development. Our study found that LRP1B was significantly upregulated in the HC group compared to the CAD and control groups, with no significant difference observed between the control and CAD groups (Figure 2C).

In contrast to recent findings, the reduced expression of LRP1B in the CAD group was significant only when compared to the HC group, and not the control group. Additionally, we observed a substantial correlation between LRP1B expression and lipid profiles across all groups (Figure 3). Based on the normal lipid profiles identified in both the control and CAD groups, we conclude that increased LRP1B expression is associated with blood cholesterol levels but is not a direct indicator of atherosclerosis.

The proto-oncogene vav (VAV) is another protein implicated in cholesterol metabolism and atherosclerosis. The discovery that VAV induces foam cell formation and the progression of atherosclerotic plaques via CD36 signaling [28] is critical for understanding the molecular mechanisms underlying atherosclerosis. CD36 is a scavenger receptor responsible for the uptake of ox-LDL by macrophages, leading to the formation of foam cells, which are a defining characteristic of early atherosclerotic lesions [28]. VAV acts as a guanine nucleotide exchange factor (GEF) that activates small GTPases, such as CDC42, which are involved in cytoskeletal rearrangement and cellular processes, including actin polymerization [29]. Together with CDC42, the role of VAV in modulating LDL-induced actin polymerization and lipid deposition in the arterial wall elucidates the intricate interplay between cellular signaling pathways and lipid metabolism in atherosclerosis [29]. In our study, the expression of VAV was significantly greater in the CAD group compared to the other groups. Furthermore, ROC analysis demonstrated high sensitivity and specificity of VAV in CAD patients, suggesting that VAV could serve as a potential indicator of atherosclerotic disease (Figure 2R). To our knowledge, no clinical studies have investigated the role of VAV in atherosclerosis. Consequently, our current findings regarding the association of VAV with atherosclerotic vascular disease in CAD patients are novel.

In addition to hypercholesterolemia (HC), inflammation is also a key player in atherosclerosis. Accumulation of ox-LDL in the arterial wall induces endothelial activation, which exacerbates the inflammatory response by releasing adhesion molecules and chemokines that mediate monocyte and T-cell recruitment [30]. Together with lipids and necrotic cells, monocytes transform into macrophages, absorb cholesterol, and become foam cells, which form the core of the atherosclerotic plaque [31]. Furthermore, the release of growth factors and cytokines promotes the migration and proliferation of VSMCs and the secretion of collagen to form the fibrous cap that covers the plaque core [32]. Plaque rupture can cause acute coronary occlusion, resulting in myocardial infarction and ischemia [33].

According to our study, SDF-1 was one of the proteins with distinct expression in CAD patients. SDF-1, also known as C-X-C motif chemokine 12 (*CXCL12*), plays a critical role in various physiological and pathological processes, including inflammation, immune cell trafficking, and tissue repair [34]. SDF-1’s association with CAD has been previously examined. For example, a genetic study identified the *CXCL12* gene as having one of the 27 SNPs associated with a higher risk of CAD [35]. Additionally, SDF-1 expression on the platelet surface was found to be significantly higher in patients with angina pectoris compared to those with other types of chest pain, suggesting a specific link to CAD [36]. Another clinical study confirmed that SDF-1 levels increase in ischemic myocardium, mediating tissue regeneration, reducing infarct size, and improving ventricular function [37].

In response to ischemia, cardiac tissue recovers from hypoxia through vascular endothelial growth factor (VEGF)-induced angiogenesis [38]. SDF-1 enhances VEGF-mediated angiogenesis by reducing ox-LDL formation and mitigating LDL’s suppressive effect on angiogenesis [39]. An *in vitro* study showed that the absence of either VEGF or SDF-1 in progenitor endothelial cells (ECs) could be compensated by the other, preserving the overall angiogenic effect [40]. Another study found that SDF-1 levels decreased in patients with angina pectoris after stent placement in the coronary artery [41]. Together, these findings suggest that SDF-1 levels are linked to tissue ischemia and decrease after reperfusion. However, they do not necessarily indicate disease severity, as plasma SDF-1 levels have been shown to be elevated in patients with CAD but do not increase further as the condition worsens [42].

Our study found a significant reduction in SDF-1 levels in CAD patients compared to the healthy control group. It is important to note that our study utilized proteomics data from previously diagnosed CAD patients and did not include individuals with acute ischemia at the time of sample collection. Therefore, elevated SDF-1 levels were not expected in our CAD group. However, the observation of lower SDF-1 expression in CAD patients compared to the control group suggests a different aspect of its role in atherosclerosis.

Actin cytoskeleton remodeling and enhanced endothelial permeability are important for maintaining vascular integrity and protecting against atherosclerosis. SDF-1 has been shown to improve endothelial barrier function and promote actin polymerization and stress fiber formation [43], which may explain the reduced levels of SDF-1 in the CAD group.

**Table 3:** Pathway analysis of significantly altered proteins in CAD and HC patients

**Pathway**	***P* value**	**Enrichment score**	**Gene name**	**Protein ID**	**Database**
*Coronary heart disease*	0.00175	30.1	APOB	APO B	DisGeNET
			APOE	APO E	
			MMP3	MMP-3	
			PLA2G7	PAFAH	
*Hypercholesterolemia*	0.0377	13.89	APOB	APO B	DisGeNET
			APOE	APO E	
			PCSK9	PCSK9	
*Cardiovascular diseases*	0.0376	15.05	APOB	APO B	DisGeNET
			APOE	APO E	
			GRK2	BARK1	
*Coronary arteriosclerosis, CAD*	0.0422	8.34	CXCL12	SDF-1	DisGeNET
			MMP3	MMP-3	
			PCSK9	PCSK9	
*Lipid and atherosclerosis*	<0.001	11.01	AKT2	PKB beta	KEGG
			APOB	APO B	
			BAD	BAD	
			CAMK2A	CAMK2A	
			CAMK2B	CAMK2B	
			CAMK2D	CAMK2D	
			CASP3	Caspase-3	
			HSPD1	HSP 60	
			LYN	LYN	
			MAP3K7	TAK1-TAB1	
			MMP3	MMP-3	
			PDPK1	PDPK1	
			PRKCA	PKC-A	
			SRC	SRCN1	
			STAT3	STAT3	
			VAV1	VAV	

Table represents the pathways that the proteins were involved in. The analysis was performed using the KEGG and DisGeNET databases utilizing the GeneCodis platform (accessible at https://genecodis.genyo.es/). HC: Hypercholesterolemia; CAD: Coronary artery disease; DisGeNET: Gene–disease associations; KEGG: Kyoto Encyclopedia of Genes and Genomes; SDF-1: Stromal-cell-derived factor-1; PCSK9: Proprotein convertase subtilisin/kexin type 9; VAV: Proto-oncogene vav; HSP 60: 60 kDa heat shock protein; TAK1-TAB1: TGF-beta-activated kinase 1 and MAP3K7-binding protein 1 fusion; APO B: APOlipoprotein B; APO E: APOlipoprotein E; CXCL12: C-X-C motif chemokine 12; MMP-3: Matrix metalloproteinase-3.

SDF-1 has also been found to be related to serum LDL levels and is elevated in HC, leading to its suggestion as a biomarker for hyperlipidemia [44, 45]. However, our results showed a nonsignificant difference in SDF-1 expression in the HC group compared to the control, which contrasts with previous findings. Moreover, we observed a significant negative correlation between TG and SDF-1 levels in the CAD group only. These findings suggest that changes in SDF-1 levels are specific to CAD and may play a role in lipid regulation and vascular health.

It is crucial to interpret these results cautiously, as they are based on a limited dataset from this study. Additional research with larger sample sizes is needed to validate these findings and provide a more comprehensive understanding of SDF-1’s role in CAD and lipid regulation. Other potential confounding factors, such as age, gender, lifestyle, and comorbidities, should also be considered in future studies. Further investigations with well-designed experimental protocols and rigorous statistical analysis are required to confirm and expand upon these findings.

This study found distinct expression of cysteine and glycine-rich protein 3 (CSRP3) and a significant negative correlation with blood cholesterol levels in the CAD group. Several genetic studies have linked CSRP3 to cardiomyopathies [46–48]; however, to our knowledge, no studies have examined its role in CAD. Thus, our finding is novel and opens new areas for research.

Our findings of upregulation of HSP 60 and TAK1-TAB1 in CAD patients compared to HC, along with their negative correlation with LDL cholesterol levels in HC patients, suggest potential roles for these proteins in atherosclerosis and dyslipidemia. HSP 60 has been previously shown to reduce lipid deposition in the arterial wall in rabbits fed a high-cholesterol diet [49]. However, an association between TAK1-TAB1 and CAD has yet to be explored.

MMP-3 is an enzyme involved in extracellular matrix remodeling, which is critical for tissue development and repair [50]. MMP-3 contributes to the breakdown of extracellular matrix components, such as collagen and elastin, which can weaken arterial walls and promote the development and rupture of atherosclerotic plaques [51]. Several studies have shown increased MMP-3 levels in atherosclerotic plaques and in the circulation of individuals with hypercholesterolemia [52]. However, our results showed no significant change in MMP-3 levels in CAD patients, indicating that further research is needed to clarify MMP-3’s role in atherosclerosis.

APO B, a protein responsible for transporting cholesterol particles through circulation, is known to be a predictive marker for CAD [53]. In our study, APO B showed notably higher expression in the HC group than in both the CAD and control groups, though it did not correlate with lipid levels in HC patients. It is important to emphasize that correlation does not imply causation. The absence of a correlation between increased APO B expression and lipid levels in HC patients may be unexpected, as elevated APO B is typically associated with elevated lipids in hypercholesterolemia. However, this discrepancy may be explained by factors, such as dysfunctional lipid metabolism or lipoprotein heterogeneity. Interestingly, we observed a distinct and significant correlation between APO B and LDL levels in the CAD group, suggesting that APO B may play a role in the development or progression of CAD. This correlation appears independent of APO B blood levels, as its overall expression was lower in CAD than in HC.

Recent studies have identified amyloid beta, cathepsin S, and negatively charged LDL as predictors of mortality risk in atherosclerosis [54–56]. Notably, studies on amyloid beta and cathepsin S used ELISA and focused on patients with non-ST elevation acute coronary syndrome [54, 55]. Our study, in contrast, does not restrict its scope based on ST elevation. Regarding LDL electronegativity, it was highlighted as a novel and independent factor for mortality risk in atherosclerotic CVD patients [56]. Their approach involved isolating LDL through sequential KBr-based ultracentrifugation and subdividing it into five electronegativity subfractions (L1–L5) using anion-exchange columns on an FPLC system [56]. In contrast, our study employed comprehensive targeted proteomics profiling using SOMAmer, providing a broader scope of analysis.

Proteomics offers advantages over traditional biomarker-focused studies by providing a holistic view of the proteomic landscape within a biological sample. This approach is invaluable for identifying potential disease risk biomarkers, as it examines the entire spectrum of proteins rather than focusing on individual markers. An overview of published proteomics studies related to CAD or HC is provided in Table S7.

This study has several limitations. While the sample size is relatively small, the number of proteins analyzed is high (*n* ═ 1305). The exploratory nature of the study underscores the need for additional research to validate data accuracy. A notable limitation is the small mean age difference between the patient and control groups. The control group has a slightly lower average age, primarily due to challenges in recruiting older, healthy blood donors. However, it is important to note that the age difference does not reach statistical significance when comparing the CAD group with the HC group (see Table S1). Despite potential age-related differences, it is noteworthy that SDF-1 and PCSK9 expression levels remain statistically significant in the CAD group compared to both control and HC groups.

The self-reported diagnosis of CAD introduces potential biases; however, the OPLS-DA model demonstrates clear group separation, with unique protein alterations in the CAD group—such as elevated PCSK9 expression—reinforcing our findings. Although asymptomatic CAD patients may exist in other groups, this does not impact the study’s conclusions. Medication usage data, including beta blockers, platelet aggregation inhibitors, aspirin, and statins (Table S2), supports the accuracy of CAD diagnoses.

Despite statistical significance, the Log2FC differences within groups may be relatively small, necessitating careful evaluation of their diagnostic potential. The clinical relevance of low Log2FC values depends on context, disease severity, and available treatment options. The OPLS-DA model’s Q2 permutation value of 0.28, though reliable, indicates room for improvement. Univariate analyses using QC plots, box plots, and ROC curves further support result validity, enhancing the study’s rigor. Validation studies are essential to confirm the potential biomarkers identified in this discovery-based proteomics study. As this study focuses on initial identification, independent cohort validation is the next critical step for assessing the reliability of these findings.

## Conclusion

Proteomics research has recently identified biological markers for the diagnosis and prognosis of CAD. Hypercholesterolemia (HC) is a major risk factor for the development of atherosclerosis. Our study identified significantly differentially expressed proteins in CAD and HC patients, with *q* < 0.05 and AUC ≥ 0.75. In total, 65 proteins displayed significant differential expression, with 22 of these proteins linked to CAD or HC-related pathways. Fourteen of these proteins were also significantly correlated with blood cholesterol levels. Specifically, the correlation between SDF-1 and APO B with blood lipids was unique to the CAD group. This exploratory study highlights potential biomarkers for CAD and HC that warrant further validation. These findings could lay the groundwork for new, cost-effective screening and risk assessment methods for atherosclerosis.

## Supplemental data

Supplementary data are available at the following link: https://www.bjbms.org/ojs/index.php/bjbms/article/view/10111/3570.

## Data Availability

Data used for this study is available online: https://doi.org/10.6084/m9.figshare.25101440
